# “You have to keep fighting”: maintaining healthcare services and professionalism on the frontline of austerity in Greece

**DOI:** 10.1186/s12939-016-0407-8

**Published:** 2016-07-26

**Authors:** Angeliki Kerasidou, Patricia Kingori, Helena Legido-Quigley

**Affiliations:** 1 The Ethox Centre, Nuffield Department of Population Health, University of Oxford, Oxford, UK; 2 Saw Swee Hock School of Public Health, National University of Singapore, Singapore, Singapore; 3 London School of Hygiene and Tropical Medicine, London, UK

**Keywords:** Austerity, Greece, Healthcare, Professionalism, Medical professionalism, Healthcare system reforms, Qualitative

## Abstract

**Background:**

Greece has been severely affected by the 2008 global economic crisis and its health system was, and still is, among the national institutions most shaped by its effects.

**Methods:**

In 2014, this qualitative study examined these changes through in-depth interviews with 22 frontline healthcare professionals in five different locations in mainland Greece. These interviews with nurses, doctors and pharmacists explored perceptions of austerity and how ideas of professionalism were challenged and revised by these measures.

**Results:**

Participants reported working conditions characterised by dramatic increases in public hospital admissions alongside decreases in personnel, consumables, materials, and also many hospital closures. Many drew on analogies of war and fighting to describe the effects of healthcare reforms on their working lives and professional conduct. Despite accounts of deteriorating conditions and numerous challenges, healthcare professionals presented themselves as making every effort to meet patients’ needs, while battling to resist guidelines which they perceived diminished their roles to production-line operatives.

**Conclusions:**

Participants considered it their duty to defend their professional ethos and serve patients without compromising standards, even if this meant liberal interpretation and implementation of regulations. These professionals regarded themselves on the frontline of healthcare provision but also the frontline defence in a war on their professional standards from austerity.

## Background

### Background to austerity in Greece

Greece is one of the European countries that have been severely affected by the 2008 global economic crisis. By 2010, the Greek deficit was calculated at 16 % of gross domestic product (GDP) and in May of the same year, Greece received its first bailout loan of €110 million from the European Commission, the European Central Bank and the International Monetary Fund, now known collectively as Troika. A second bailout of €130 million followed in February 2012. A third loan of up to €86bn to be paid in several instalments was agreed in August 2015. These loans came with strict conditions regarding implementation of austerity measures, structural reforms and privatisation of government assets. The period since 2010 has been characterised by great political and social instability, as successive governments try to implement the measures and structural reforms required by the Troika. Since 2010, Greece have had seven different governments, including an unelected technocratic government (November 2011 to May 2012) and two interim governments, and held national elections four times (May 2012, June 2012, January 2015 and September 2015).

### Austerity and health

The Greek healthcare system was among the national institutions affected by the reforms and austerity measures. Between 2010 and 2014, the healthcare system was restructured twice, aiming at increasing its efficiency and also meeting Troika’s directives, which necessitated the reduction of public spending as a condition for the release of funds. The first restructure came in 2011, which merged the pre-existing social and health insurance funds into a unified central health fund (EOPYY: National Organisation for Healthcare Provision) to decrease the burden on the state [1]. The Greek healthcare system saw further restructures in 2014, which established a new National Primary Healthcare Network (PEDY) [2]. One of the Troika directives was that public expenditure on health should not exceed 6 % of GDP [3]. This measure produced a 30 % reduction in hospital budgets [3], a 40 % cut in healthcare professionals’ salaries [4], and a 10–40 % reduction in staff [4]. In addition, cancer screening programmes [5], mental health services, prevention and treatment programmes for illicit drug use, and municipal public health services experienced mass closure of services and severe cuts [3, 6].

The cuts in healthcare spending coincided with deterioration in the Greek populations’ average income due to reductions in salaries, pensions and benefits, and rapid growth in unemployment. Between 2008 and 2013 official unemployment reached 29.4 %, and 51.1 % of young people were unemployed in the last quarter of 2014 [7]. The severe material deprivation rate, which measures the proportion of people with living conditions characterised by severe lack of resources, exceeded 20 % in 2013 [8]. These factors meant that many turned from the private to the public health sector, at a time when the latter was experiencing the aforementioned cuts in resources. Between 2009 and 2010, public hospital admissions grew by 24 %, with a further rise of 8 % in the first half of 2011, while private hospital admissions decreased by 25–30 % [4].

The year 2011 saw the increase of co-payments for medicines and services, and the introduction of user-fees for outpatient visits. As unemployment increased, a large proportion of the population lost their health-insurance coverage [9]. In 2013, as a result of public pressure, a health-voucher scheme was introduced, which offered uninsured individuals limited to access primary healthcare (PHC) services [3]. In 2014, further changes were introduced to allow for the uninsured to access health services and medicines. However, these schemes have been of limited success [10].

### The effects on healthcare professionals

Healthcare professionals were also affected by austerity measures. They suffered salary cuts of 15 %, a 10 % cut in pensions, abolishment of additional monetary bonuses, while their retirement age has increased from 65 to 67 [4]. There has been a paucity of research exploring the experiences of healthcare professionals. What exists examines, at hospital level, emotional burnout among staff and medical supply shortages [11]; how occupational factors and economic crisis affect smoking in health professionals [12], and under-the-table informal payments [13]. At PHC, there are two qualitative studies exploring the perception of healthcare professionals on the quality of services in rural areas [14, 15]; and one in an urban setting [16]. Studies have explored the perceptions of Greek medical students regarding the medical profession and the speciality selection process during the financial crisis [17]; physicians’ brain-drain as a result of austerity measures [18]; and broader discussions around the healthcare professional’s role in the provision of services in times of austerity [19].

This paper is distinctive as a qualitative examination focusing on the experiences of healthcare professionals in Greece. It aims to provide insights from the views and positions of frontline healthcare staff in austerity Greece at different levels of the system. This focus includes how they have experienced austerity measures; the types of ethical dilemmas faced; their obligations and responsibilities; and also, efforts to manage expectations from patients and patients’ families. This paper also aims at providing a more detailed analysis of how these measures are affecting healthcare professionals’ ability to cope, and explores how their idea of professionalism has been challenged and revised in the face of austerity. Their views and positions are examined and discussed, after presenting the methodological tools and data collection strategies used in this study.

## Methods

The central question of this study was: *In what ways has the financial crisis, and subsequent austerity measures, affected the everyday ethical decision-making and experiences of frontline healthcare professionals?* Given its aims and focus, a qualitative approach was adopted to allow for a nuanced understanding of the lived experience of austerity among healthcare professionals in Greece. The main method was semi-structured interviews. The interview-guide was broadly informed by the literature and media coverage of the effects of austerity on health in Greece, and sought to capture professionals’ views on this issue. The interview-guide was devised by PK and AK. AK conducted all the interviews in Greek in two instalments between March 2014 and December 2014. To maintain confidentiality, the interviews were transcribed and translated into English by individuals not professionally associated with the Greek healthcare system, and not aware of the interviewees’ identities.

### Sampling

This study was undertaken in five different locations in mainland Greece, which included urban centres, towns and villages to afford a wide geographical and population coverage.

This study employed two methods to sample participants. The first was purposive sampling, selecting respondents by their geographic location (rural and urban), level of seniority (e.g. registrars, consultants), employment role (nurses, doctors, pharmacists) and employment status (e.g. self-employed, private or public). The second approach involved a snowball sampling technique, where healthcare professionals were asked to nominate others to share their views and experiences.

In total, 22 semi-structured interviews were undertaken with frontline healthcare professionals (nurses, doctors and pharmacists) from urban and rural locations in mainland Greece. Table 1 presents the study participants by title and type of healthcare facility in which they worked.

**Table 1:** Study participants by title and type of healthcare facility

	Public hospital health centre clinic	Private hospital clinic	Self-employed	Self-employed + EOPYY	Self employed + EOPYY until >12 months ago	Total
Doctor	10	1		3	3	17
Nurse	2	1				3
Pharmacist			2			2
Total	12	2	2	3	3	22

The interviewees were purposively sampled based on their position at the frontline and within the selected locations. The place and time of the interview was dictated by study participants. Interviews were conducted at their place of work, in public places (e.g. café) or at their home, and were between 30 to 90 min long. A factor that influenced the length of the interview was the place it was conducted. When carried out at their work-place, interviews where kept short to minimize the impact on their work-day. Interviews that took place outside the work-place where much longer. Many participants mentioned that they found the interview process interesting and even therapeutic, as they felt it was the first time someone was interested in their views and experiences.

### Analysis

AK and PK coded all interviews through an inductive approach and thematic analysis drawing on techniques from the constant comparative method, such as line-by-line analysis of early interviews, naming each line and segment of data, and identifying deviant cases [20, 21]. The data was managed using QSR NVivo 10 Software. The data was discussed throughout by AK, PK and HLQ.

## Results

The data collected in this study are thematically presented in two parts. The first section describes healthcare professionals’ perception of how they have been affected by austerity measures. It focuses on micro-level experiences of austerity including views on salary reductions, staff shortages and increased admission in public hospitals. The second section explores the influence of austerity measures on concepts and views of professionalism among participants. We present the patient-professional relationship and austerity-related ethical dilemmas as the two main ways that professionalism in these interviews discussed by participants. The paper is underpinned by war metaphors used by participants to describe the effects of austerity.

Table 2 provides a summary of the themes and subthemes presented and discussed below.

**Table 2:** Summary of themes and subthemes

Theme	Subtheme	Representative quote
Personal experience of austerity measures in providing healthcare services	Increasing admissions in public hospital and decreasing resources	*“The number of patients coming into the hospitals has risen significantly. The people have started using public hospitals more the past few years […] they decide to come straight to A&E.” (I11)*
	Diminishing Access to treatment and medication	*“In the past people used to pay their contribution in full and on time. Now there is no access to medication, not because of increased prices for the medication – medication have had more than twenty price drops in the last two or three years according to official sources – but the patient contribution has been increasing constantly.” (I19)*
Austerity and ideas of professionalism	Disregarding the profession: feeling disvalued and disrespected	*“When someone is in the operation room and you pay them €4 per hour to save a life, it’s like you insult their work, there is no question about it. And I should better leave it at that. You just insult what they do.” (I8)*
	Challenges to professionalism	*“You cannot provide the same care that you want as a doctor and that the patient needs. […] what suffers in this situation is the conversation with the patient. You just don’t have time to discuss, to listen to the patient a bit more.” (I4)*
	Efforts made to maintain professionalism	“*This means that regardless of how tired I feel, regardless of how my patient will talk to me, I have to respect them. Yes, by giving a big battle that has a personal cost, I believe that I provide my patients the best I can as a doctor. I really believe that I do my best at least as far as my job is concerned and that I haven’t allowed my standards to fall”. (I3)*
	Professionalism and Ethics	*“This is what I mean by swindles. We come up with solutions like this one with the hope to save €5, €10. Even these €5 and €10 are quite important to someone who does not have a job”. (I19)*

All interviewees perceive that there was a need for reform in the Greek healthcare system, e.g. rationalisation of prescriptions and laboratory tests, and where aware of its inadequacies and problems. However, most professionals suggest the austerity measures and subsequent healthcare reforms are not addressing what they regard to be the core issues but, rather, the new measures often exacerbate problems for professionals and patients. The forthcoming sections discuss the reasons for reservations among frontline healthcare professionals by presenting their experiences of working within austerity-driven healthcare reforms.

### Personal experience of austerity measures in providing healthcare services

The first key theme that emerges from the data describes healthcare workers accounts which liken the effects of austerity measures on healthcare services to *“being in a war-zone”*. This theme is divided in two subthemes: (1) Increasing admissions in public hospital and decreasing resources; and (2) Diminishing Access to treatment and medication. It presents personal accounts from participants about how their daily experiences are affected, and provides insights into the circumstances in which nurses, doctors and pharmacists find themselves on the frontline of the effects of austerity on healthcare.

#### Increasing admissions in public hospital and decreasing resources

The frontline healthcare professionals interviewed provide personal insights of living and working in Greece with aforementioned official statistics. One doctor working in a large public hospital describes her experience of the growth in public hospital admissions:

The number of patients coming into the hospitals has risen significantly. The people have started using public hospitals more the past few years […] they decide to come straight to A&E. […] **A night-shift in (Name of Hospital) is like being in a war-zone! Proper war!** [Emphasis added] (I11).

The analogy of being in *“war-zone”* is used frequently during interviews with healthcare staff to describe their working conditions, and also the type of strategies they feel it necessary to adopt to manage the effects of austerity on healthcare services. They regard services as being under assault from the cumulative effects of increasing admissions to public hospitals and decreasing staff, hospital budgets and salaries. A microbiologist based at another public hospital explains his large workload as follows:

…our hospital is on-call four days a week. Previously, when the hospital was on-call we had six microbiologists working. The last six months, the administration has reduced the number to four […] when it is only four, we find it difficult because the workload is big (I1).

The effects of staff shortages are compounded by drastic reductions in consumables and materials. In the following quote, a hospital surgeon describes what a 30 % reduction in hospital budgets means in practice for those on the frontline. She divulges that this involves having to economise on tools and stitches:

In surgery some things are being done manually, although they could be done with a specialized tool. Or we have limited number of tools. **We have 20, for example, while we need 40, so we have to economise**. Or we don’t have the right stitches (I8) [Emphasis added].

#### Diminishing access to treatment and medication

Most interviewees reported the opinion that the biggest and most pressing problem faced by the Greek population is less about access to doctors but more about access to *treatment and medication*. One pharmacist explains that this is not due to the increased price of medication but rather the escalation in price of co-payments, which have transferred most of the cost of medication from the state to individuals. He states that:

In the past people used to pay their contribution in full and on time. Now there is no access to medication, not because of increased prices for the medication – medication have had more than 20 price-drops in the last 2 or 3 years according to official sources – but the patient contribution has been increasing constantly (I19).

Therefore, rather than patients having easier access to medication, it has become much harder to afford them. In what follows a doctor, volunteering in a Community Clinic, which relies on donations to provide healthcare and treatment to those unable to afford it, explains how austerity measures has shaped access to medicines:

…the problem is that they cannot afford to buy their medication… In the end of the day, someone will always find a doctor to examine them; they will either go to a public hospital and pay these €5, which is not much – of course nowadays there are people who cannot even afford to pay these €5- or they can go to a regional clinic which is free, or **there are many doctors, good people who see many patients for free, I personally know many doctors who do that. But no one will ever give them their drugs for free** (I13) [Emphasis added].

This quote provides insight into the financial circumstances of many Greeks who cannot afford healthcare costs. Another insight which can be gained from this quote is one of the strategies on the war on austerity-driven healthcare reform, which are adopted by frontline healthcare workers such as the doctor interviewed. Despite their increased workload and reduction in incomes many of those interviewed, conscious of the causalities of these reforms, treat patients without charge.

### Austerity and ideas of professionalism: “You have to keep fighting”

The second part of this paper explores how austerity measures affect professional identity and professionalism, and is divided in four subthemes. These are: (1) Disregarding the profession: feeling disvalued and disrespected; (2) Challenges to professionalism: “you catch yourself becoming inhumane”; (3) Efforts made to maintain professionalism: “I haven’t allowed my standards to fall” and (4) Professionalism and ethics: “we do the right thing” versus what they are being asked to do.

#### Disregarding the profession: feeling disvalued and disrespected

The majority of the interviewees recognise that because of the country’s critical financial situation, difficult decisions have to be made. They also acknowledge that it was not only the healthcare sector that was suffering from austerity-driven policies: *“our country is strangled by its debts and responsibilities to Troika; all sectors are bad, industry, agriculture, everything!”* (I17). Yet, many frontline healthcare workers feel strongly that the austerity-driven reforms not only cause them financial hardships, but undermine them as professionals. The following doctor argues that it is not only patients who are the casualties of these reforms but the respect and value shown to doctors has suffered too. He explains how the low salaries offered to new doctors are interpreted as an affront to their profession:

When you don’t hire new doctors on the islands or in other remote areas, and there are so many residents there without a doctor and you do nothing about it. When you send someone with €1000 salary (on a 1-year fixed-term contract), someone who is 35–40 years old, who probably has a family, how can you send this doctor for 1 year to this island? You could at least have them stay there for 5 years, isn’t that right? At least you could give them some time to get there, to find a house, to settle in that place. So it is as if they are mocking us. They are mocking both the doctors and the patients (I8).

For such reasons, many frontline healthcare professionals interviewed regard their allegiances as being with their patients, as both groups are perceived to be under attack by austerity measures. All of those interviewed mention that the drastic reduction in their salaries impacts on how they view themselves and their profession. Here, an interviewee gives an account of what this income reduction means for existing frontline healthcare staff:

[O]ur salary has been reduced by 50 %, in comparison to the salary we used to get before the financial crisis. And **although we do many things out of […] the kindness of our heart, because we do not want to leave our patients without care, without treatment, without medications, this reduction in our income is very disheartening and disappointing** (I12) [Emphasis added].

As stated earlier, one strategy in the war on austerity-driven healthcare reforms is to maintain pre-existing levels of patient care and treatment despite the obstacles. However, the effects of austerity-driven healthcare reforms, as described above, combined with the dramatic reduction in their income, leave many frontline professionals feeling disvalued and disrespected. Being forced to work under such conditions, many feel, demonstrate that government proposals lack understanding of the healthcare profession, but also lack of interest for the welfare of health professionals and patients alike. The following quote makes this view clear:

When someone is in the operation room and you pay them €4 per hour to save a life, it’s like you insult their work, there is no question about it. And I should better leave it at that. You just insult what they do (I8).

This idea of being insulted and affronted is a theme repeated by those interviewed, on the topic of salary reductions. In general, the huge reduction in earnings is deemed to be an affront to the whole profession, which undermines motivations and demeans the importance to their work. In the following quote, we learn from a healthcare profession why doctors are deserving of respect:

… **the political system regards the doctor as just a service provider, the same as any other public servant. Yet, from the moment that they regard them as that, they seize to respect them**. So they will assign to them a thousand jobs, without paying them properly, and even worse than that, without showing any respect…a doctor deserves respect […] because they put their own health in danger for the sake of their patients’ (I3) [Emphasis added].

In this quote, it is the political system and the Greek State which is perceived to have waged war on healthcare professionals. One doctor admits that she would have been happy with her current salary, if her work schedule was less physically, mentally and psychologically overbearing *“I wouldn’t mind getting less money, if the situation was less stressful, honestly!”* (10). The stress experienced by frontline staff is described as effecting working conditions, views of reforms and also challenging their ideas of professionalism.

#### Challenges to professionalism

A situation characterised by high demands and low returns creates concern that eventually competent doctors with experience would leave the public health system. For many, this is not perceived as a sign of disregard for the social value of their profession, but mainly as a sign of self-preservation in this ‘war-like’ situation. This interviewee explains further that:

**…doctors are also human, and the doctor will also think, why am I working like a dog all day for 24 h only to get paid €60 in the end? **[…] think about it, the doctor only gets €60 for being on-call all night, and the clinical doctor is dead-tired and stressed […]’ (I1) [Emphasis added].

Furthermore, doctors and nurses felt that the situation was distorting what they perceived to be the character of their profession. An A&E nurse describes her experience as follows:

Now regarding the A&E, when you see them (the patients) stacked, literally, three times the number we can handle … a dignified examination needs some time, you need to check their health history, to do the clinical examination… **I have the feeling that we work in a factory. Each patient has 5 min, when one goes another comes in** (I6) [Emphasis added].

These quotes provide valuable insights into what is perceived to be some of the major challenges to professional ethos of those frontline healthcare professionals who remain in the public health system, namely; insufficient pay and an inability to care for patients in a way they consider appropriate and dignified. This doctor discusses how insufficient time for discussion impacted his treatment of cancer patients:

**You cannot provide the same care that you want as a doctor and that the patient needs. […] what suffers in this situation is the conversation with the patient. You just don’t have time to discuss, to listen to the patient a bit more.** Because it is not possible to announce to a patient that they have cancer and not give them some more time for discussion […] Due to this heavy workload…there is no option of delivering such an announcement privately to the patient (I4) [Emphasis added].

Ultimately, there are fears that although, many professionals are undertaking duties which are over-and-above the guidelines, the stress of their working conditions would undermine their professionalism and produce inhumane practices towards patients. This interviewee gives further details of this point:

It (your profession) does not represent you anymore, because you catch yourself becoming inhumane. Because when you stop saying ‘hello’ and ‘goodbye’ or anything beyond the necessary care, a big part of nursing as a holistic care stops to exist’ (I5).

#### Efforts made to maintain professionalism: “I haven’t allowed my standards to fall”

Despite the difficulties experienced in conducting their everyday duties, frontline healthcare professionals in Greece emphasise the efforts they make to maintain high standards within the parameters of the available resources. It is felt by those interviewed that these efforts often come at great personal cost; at the expense of their time with their family, holidays and days-off. Many mention that the accumulation of these factors result in physical and psychological stress and burn-out. The following quote extends the war analogy presented earlier in the paper. The healthcare professional describes their attempt to maintain high-standards of service to patients as a form of resistance, a fight and a battle in the face of these cuts:

A big group of health professionals resists these changes and insists on offering a service, possibly, as good as it used to be before the crisis….**you have to keep fighting so that you don’t let them (the changes) affect your work**. In my case, personally, it hasn’t been affected. But it hasn’t been affected in exchange with a specific psychological cost, right? Across my desk, I have a piece of paper facing me, the patients cannot see it, which reads ‘Respect’. This means that regardless of how tired I feel, regardless of how my patient will talk to me, I have to respect them. Yes, **by giving a big battle that has a personal cost, I believe that I provide my patients the best I can as a doctor. I really believe that I do my best at least as far as my job is concerned and that I haven’t allowed my standards to fall** (I3) [Emphasis added].

This quote reveals a number of important aspects of how the changes in the healthcare resources available have shaped the everyday practices for frontline staff. It demonstrates that the fight and resistance mentioned among staff interviewed is for the patients’ benefit. As this interviewee discussed, the current cuts placed staff in situations where they need to fight not allow for “standards to fall”, regardless of personal and psychological costs. For those interviewed, this is their way of enacting professionalism and it is this, in addition to maintaining a respect for patients, which they feel is under attack.

#### Professionalism and ethics: “we do the right thing” versus what they are being asked to do

Frontline healthcare professionals use various ways to maintain their sense of professional ethos, whilst attempting to adapt to the changes brought by the financial crisis and the new structures in healthcare. As discussed above, dramatic increases in unemployment led to an increase of the population without health insurance, which severely restricted their access to healthcare. For frontline healthcare professionals, managing requests from the uninsured raises tensions between following the rules and fulfilling their duties as doctors and nurses. In these instances, the staff interviewed mention that they manage such tensions and dilemmas by interpreting the new rules loosely, to make them comply with their sense of professional duty. In this quote, this doctor explains the importance of doing “the right thing” by all of their patients, irrespective of whether they have health insurance or not.

Our guidelines are that we have to medically treat them (the uninsured) as long as it is an emergency, and this is certainly what happens. As for the chronic conditions, things are bit muddled. For these situations the guidelines are vague and their implementation tends to be more towards trying to cover them [the patient] medically, although this means that we could face some sort of repercussions, but admittedly, this does not happen. […] But there are people who suffer from kidney failure, and need dialysis… for this person to be considered an emergency case they must have reached a pre-death stage […] On the other hand providing them with a dialysis three times every week would be considered chronic treatment. Well, **we label the dialysis as an emergency treatment and we go for it. And nobody stops us. And of course we do the right thing** [Emphasis added] (I9).

The example given here of rule bending and loose interpretation of guidelines is mentioned as an important strategy in reconciling the differences between what healthcare professionals feel they are being asked to do, versus what they feel is the right thing to do. When it comes to the actual treatment of patients, they feel that some of the rules and regulations introduced are contrary to effective clinical practice and patient management. This, they feel, makes their everyday working lives difficult.

This quote provides a further example of how doctors are trying to adhere to the new regulations, whilst striving to do the best of their patients.

So, if you want to do a general check-up, you need more than 5 tests (a doctor can only prescribe maximum of 5 tests at a time for a patient).... The tests for a mini check-up amass to at least 10. So, you will say to the patient, I need these test, but I cannot prescribe them all. Can you pay for the rest?. And they will say: Doctor, I am unemployed, etc. etc. So, what we do is check the list and see which ones are the most expensive, and I will prescribe those, so at least the patient will have to pay out of his own pocket for the cheaper one. **This is what I mean by swindles. We come up with solutions like this one with the hope to save €5, €10. Even these €5 and €10 are quite important to someone who does not have a job.** I see patients, respectable people… who would come and ask me: ‘Doctor, please help me…I cannot afford to pay for all these. Both I and my wife are unemployed. We all live off my father’s pension.’ So, what can I say to this person? He looks at me in the eyes and tells me these things. What can I say to this person? So, that’s the situation. (I19) [Emphasis added].

The next quote is an extract of an exchange between AK(I) and a surgeon (DR) commenting on the guidelines regarding treating the uninsured. The surgeon confirms that doctors place the welfare of patients before formal rules or financial cuts. The discussion unfolded as follows:

I: Has there ever been a case when you had to deny treatment to someone, after dealing with any urgent issues of course?’

DR: No, never.

I: Never in all the years you have been working?

DR: No, you cannot do this.

I: Why can’t you do it?

DR: Well, it’s an issue of our professional code of ethics, how can you do this?

I: And what about the management? What if the management told you that there is no money to cover for this patient?

DR: Then it is for the management to find a solution, and not me (I8).

This extract provides an explicit and valuable account of what constitutes a professional code of ethics. It is important for healthcare professionals that their standards are never compromised and they are defiant of rules and regulations they feel directly contradict their professional ethos.

## Discussion

From the data presented in this paper, it is clear that the financial crisis and austerity measures have affected healthcare in Greece. The frontline healthcare professionals reported that they were operating in an understaffed, underfunded and mismanaged healthcare system, where they are expected to work many more hours under difficult conditions and see more patients for less pay. These findings resonate with publications on the effects of austerity cuts on services’ demand and service provision. It has been reported that rural areas have particular difficulties with increasing shortages in drug and medical equipment supplies [14]; and with the increasing numbers of patients who now seek public rather than private healthcare services [16]. Other concerns raised include the necessity to address the healthcare needs of uninsured people [15].

Another study exploring the frequency of medical supply shortages and their possible impact on burnout risk of healthcare workers, concluded that such shortages were significantly associated with emotional exhaustion and depersonalization among healthcare workers [11]. This account supports the findings in our study.

Similar research conducted in Spain also identified healthcare professionals working over-time to maintain the quality of services provided. Participants in these studies explained that working conditions had worsened due to reduction in salaries and cutbacks to substitute hiring, with most participants reporting burnout as a result [22, 23].

In this study, participants argued that the old healthcare system was in need of restructuring. They mentioned that there were systemic inefficiencies that required immediate attention and reform, such as rationalisation of prescriptions and laboratory tests. However, in practice, austerity-driven healthcare reforms challenged frontline doctors’ and nurses’ idea of professionalism. In the philosophical and bioethical literature, theories of professionalism are described as guides individuals use to identify the role and purpose of their profession in society. They act as moral compasses, by revealing fundamental ethical virtues and principles that should govern a profession, and by steering individuals towards the right action [24, 25]. Although, basic principles and virtues of professionalism seem to remain unchanged, the ways in which they are practiced are influenced by the environment and circumstances in which professionals find themselves [26, 27]. As Tallis notes, ‘professionalism acts as the continuity and counterweight to changes in policies concerning health care delivery that can sometimes strain services and sometimes introduce new uncertainties into patient care’ [26]. Our findings confirm these theoretical expositions of professionalism. From the accounts presented here, perceptions of professional identity and professional duties served as crucial guides to frontline healthcare professionals and assisted them in devising solutions to the practical and ethical problems they encountered.

When healthcare professionals interviewed as part of this study were faced with ethical dilemmas, their professional code of conduct provided them with guidance towards what they felt was the right answer. For example, managing requests from uninsured persons raised tensions between following the new rules and fulfilling their duties as doctors and nurses. In these instances, those interviewed mentioned that they managed such dilemmas by interpreting the new rules through the lens of their professional ethos. This finding also corresponds with experiences in Spain, where the Spanish government has excluded undocumented migrants from the healthcare system, and Spanish healthcare staff reported exercising conscientious objection to the new regulation and continuing to serve this population in the same manner as before [22]. However, many of those interviewed in this study in Greece expressed concerns regarding whether healthcare professionals would be able to retain their professional ethics and ethos in these austere and adverse climate for much longer.

Regarding professional identity, our study showed that professionals perceived the salary reductions and deterioration of their working conditions as an affront to their sense of dignity and worth. There is an extensive literature linking employees’ work-related behaviour and motivation with the salary received [28]. Other studies show the connection between a sense of self-worth, feelings of being valued by the employing organisation and levels of compensation [29, 30]. Gardner et al. propose ‘that pay level signals employee worth to the organization and influences beliefs about personal adequacies and worthiness as an organizational member which, in turn, influence employee performance’ [31]. It has been suggested that when a salary reduction is necessary, for example in situations of adverse economic conditions, employers should use other methods to signify to their employees that they are still valued [31, 32]. Yet, healthcare professionals in Greece felt disvalued and disrespected, and they saw the salary and staff reductions combined with increasing service demands and unworkable reforms as a direct insult to the social role and value of their profession.

The overall findings of this study are that frontline healthcare professionals reported that they felt their working conditions was “like being in a war zone”, but that they “have to keep on fighting” for the patients’ benefit. Despite many constraints, participants still reported finding the resolution and motivation to take care of patients, adhere to their professional ethos, treat them with respect, and provide good quality services.

### Strengths and limitations of the study

This paper is distinctive in discussing the experiences of healthcare professionals from the perspective of how it affects their professionalism. The strength of this paper is that it highlights how reforms and structural changes in healthcare are lived by the people who are tasked with enacting them, namely the healthcare professionals. Through in-depth interviews, we have elicited accounts of the role of professionalism in providing justifications and guiding action.

However, this paper lacks data from healthcare professionals working in remote areas of rural Greece, such as remote islands or in mountainous areas. Although, we purposefully included participants from rural areas, these are still well-connected to big urban cities. Many of our interviewees mentioned that although they were experiencing great shortages in materials and staff, they expected that these problems would be greater in more remote areas of the country. This paper also lacks insights from unemployed healthcare professionals, or other professionals involved in healthcare, such as hospital managers and administrators, who might have experienced the austerity measures differently. However, given the aims and resources of this study it was not possible to include these groups.

## Conclusion

Frontline healthcare professionals were struggling to maintain the same level of professionalism in their everyday practice in the changing and challenging circumstances of austerity-driven health reforms in Greece. They expressed concern about their patients, and emphasised their resolution to always provide the best care possible. However, they acknowledged that increases in patient numbers using the public healthcare system and the simultaneous reduction in staff is affecting the way healthcare is practiced. The increased workload, some of our interviewees maintained, has turned the medical profession into a production-line job. Also, they felt that the significant decrease in pay has insulted their profession. Feelings that their role in society and their hard work is not acknowledged by the state were common among those interviewed. They argued that they were being attacked by having greater demands placed on them without adequate consideration for their welfare or that of their patients, which resulted in a sense that their profession was being degraded and devalued.

Despite the deteriorating situation they encountered, frontline healthcare professionals were still trying to adhere to the ideals and ethos of their profession. In practice this meant that they acted to resist the role of the production-line worker, they bent rules to accommodate the values of holistic care to patients and they were humane in the face of restrictive rules and regulations. However, these efforts often came at a high personal cost, and participants talked about feelings of stress and burnout.

Our research findings are particularly interesting for healthcare system officials as they clearly demonstrate the effects and implications healthcare reforms have on the ground, and more specifically, from the perspective of those tasked with enacting and implementing them. They also reveal that healthcare professionals are not opposed to reforms and change; rather they fight against measures they believe risk patient safety and conflict with their professional ethos. It is important for healthcare officials to be aware of the challenges healthcare professionals face and of their concerns, as it could help them structure public health systems that are sustainable, effective and beneficial for the public as a whole.

## Abbreviations

A&E, accidents and emergency; AK, Angeliki Kerasidou; CUREC, Central University Research Ethics Committee; EOPYY, National Organisation for Healthcare Provision; GDP, gross domestic product; HLQ, Helena Legido-Quigley; PEDY, National Primary Healthcare Network; PHC, primary healthcare; PK, Patricia Kingori
